# SERS-Active Substrate with Collective Amplification Design for Trace Analysis of Pesticides

**DOI:** 10.3390/nano9050664

**Published:** 2019-04-27

**Authors:** Jaya Sitjar, Jiunn-Der Liao, Han Lee, Bernard Haochih Liu, Wei-en Fu

**Affiliations:** 1 Department of Materials Science and Engineering, National Cheng Kung University, 1 University Road, Tainan 701, Taiwan; jaya.sitjar@gmail.com (J.S.); rick594007@hotmail.com (H.L.); hcliu@mail.ncku.edu.tw (B.H.L.); 2 Medical Device Innovation Center, National Cheng Kung University, 1 University Road, Tainan 701, Taiwan; 3 Center for Measurement Standards, Industrial Technology Research Institute, No. 321, Kuang Fu Road, Sec. 2, Hsinchu 300, Taiwan; weienfu@itri.org.tw

**Keywords:** surface-enhanced Raman scattering, gold nanoparticles, zirconia nanopores, pesticide detection

## Abstract

Health risks posed by the exposure to trace amounts of pesticide residue in agricultural products have gained a lot of concerns, due to their neurotoxic nature. The applications of surface-enhanced Raman Scattering (SERS) as a detection technique have consistently shown its potential as a rapid and sensitive means with minimal sample preparation. In this study, gold nanoparticles (Au NPs) in elliptical shapes were collected into a layer of ordered zirconia concave pores. The porous zirconia layer (*p*ZrO_2_) was then deposited with Au NPs, denoted as Au NPs (*x*)/*p*ZrO_2_, where x indicates the deposition thickness of Au NPs in nm. In the concave structure of *p*ZrO_2_, Au-ZrO_2_ and Au-Au interactions provide a synergistic and physical mechanism of SERS, which is anticipated to collect and amplify SERS signals and thereafter improve the enhancement factor (EF) of Au NPs/*p*ZrO_2_. By taking Rhodamine 6G (R6G) as the test molecule, EF of Au NPs/*p*ZrO_2_ might reach to 7.0 × 10^7^. Au NPs (3.0)/*p*ZrO_2_ was then optimized and competent to detect pesticides, e.g., phosmet and carbaryl at very low concentrations, corresponding to the maximum residue limits of each, i.e., 0.3 ppm and 0.2 ppm, respectively. Au NPs (3.0)/*p*ZrO_2_ also showed the effectiveness of distinguishing between phosmet and carbaryl under mixed conditions. Due to the strong affinities of the phosphoric groups and sulfur in phosmet to the Au NPs (3.0)/*p*ZrO_2_, the substrate exhibited selective detection to this particular pesticide. In this study, Au NPs (3.0)/*p*ZrO_2_ has thus demonstrated trace detection of residual pesticides, due to the substrate design that intended to provide collective amplification of SERS.

## 1. Introduction

Trace detection of contaminant or target substance has gained a lot of interests to be effective for various applications, including food safety, environmental monitoring, and medical diagnostics among others [1]. In spite, analytical methods for such applications have already been established, for example, the techniques of chromatography [2] and mass spectroscopy [3]. The said methods are mostly suitable for cases wherein the analyte component in a sample is present at a relatively high concentration, and could also follow a time-consuming process for sample preparation.

Pesticides are undoubtedly effective in minimizing pest infestation on agricultural products, thus preventing losses in product yield. However, there has been a growing public concern on the residuals of pesticides being present in agricultural products intended for consumption and eventually posing health risks. Regulations have thus set maximum residue limits for the residuals of pesticides in products; however, these limits may range from 0.01 to 0.1 ppm, which would require a sensitive detection method [4]. In particular, a fast and precise technique is needed.

The technique of surface-enhanced Raman scattering (SERS) has demonstrated its versatility in rapidly detecting various types of molecules in a straightforward process, requiring a few to no sample preparation at all, while still maintaining integrity and accuracy of presented results. A SERS-active substrate is usually comprised of plasmonic metal nanostructures (e. g., Au, Ag, Cu) since the localized surface plasmon resonance (LSPR), which amplifies the characteristic Raman signals of the analyte(s) arises from collective oscillations of free electrons in these nanostructures [5,6]. Signal enhancement in SERS is based on two mechanisms–electromagnetic (EM) and chemical mechanism (CM). The former has the major contribution to the resulting SERS effect and particularly depends on the presence of localized hot spots between nanostructures where EM field resonance take place, following by interacting with the analyte species that are in close proximity (<5 nm) with the said nanostructures [7]. The EM mechanism relies largely on the physical features of the substrate (e.g., nanostructure geometry and materials for substrate components) and therefore it can be purposely tuned. Aside from substrate’ morphology, the choice of Raman laser wavelength and resulting peaks enhancement can be optimized. [8] Recently, studies have incorporated dielectric materials and oxides, such as TiO_2_, ZrO_2_, and Al_2_O_3_ alongside Au and Ag nanostructures for further SERS signal enhancement [9,10] to facilitate charge transfer with plasmonic nanostructures in relation to CM. Mechanisms explaining the transfer of active metallic electrons to the dielectric material states that localized EM fields could be formed at the metal-dielectric interface, due to charge transfers [11,12,13]. However, in terms of fabrication, metallic and dielectric components take on different synthesis routes, so to be able to produce SERS-active substrates that make use of both of these materials, multiple fabrication techniques would be necessary [14]. In particular, a zirconia (ZrO_2_)-based surface has demonstrated an extensive SERS contribution as a component of a metal-dielectric hybrid structure. It provides additional enhancement of SERS effect, not only comes from the charge transfers at the metal/dielectric interface, but also due to a bulge or concave structure serving as high-surface area support; presumably it may accommodate more plasmonic nanoparticles (NPs) on the surface [15,16]. ZrO_2_ is also capable of anchoring specific functional groups to its oxide surface owing to strong affinities with a variety of chemicals, so this property could be taken as an advantage to tailor the functionality of the surface and improve adsorption with specific target analytes [17].

SERS as a technique for pesticide detection has been cited in several studies, and has shown promising results to be a rapid method that could even detect analytes at trace amounts. Several materials and morphologies have been explored with this particular application in mind—Ag nanoflowers [18], Au/Ag nanorods [14], and colloidal Au nanoparticles [19] among others, all of which showed capabilities of detecting low pesticide concentrations, down to 10^−10^ M. However, aside from sensitivity, there are still more factors to consider in this particular application, such as the reproducibility of results and the capability of the substrates to detect multiple pesticides in real food matrices [20,21]. To address these issues, morphology control and material selection have been given emphasis, focusing on achieving large-scale uniformity in obtaining SERS measurements [22].

In this study, a straightforward method for substrate fabrication was performed wherein ZrO_2_ with the concave structure was formed through sol-gel technique and assisted by a template of single-layered polystyrene PS NPs after which, Au NPs were deposited through thermal evaporation. The as-prepared substrate was designed to investigate both the individual and combined SERS effects from the synergy of Au and ZrO_2_ components. Its specially-designed function as collective amplification of SERS effects was verified by trace analysis of pesticides and its selectivity towards organophosphates. The optimized SERS-active substrate was furthermore applied for the distinction of residual single and multiple pesticides at very low concentrations.

## 2. Materials and Methods

### 2.1. Fabrication of Au NPs (x)/pZrO_2_

**Figure 1:**
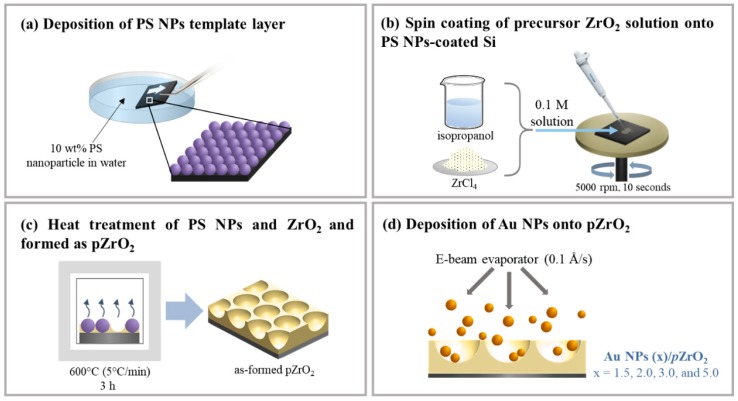
Schematic illustration of the fabrication of the Au NPs/pZrO_2_ surface-enhanced Raman Scattering (SERS) substrates: (**a**) Formation of the pore template through deposition of monolayered polystyrene nanoparticles onto the Si substrate; (**b**) deposition of a precursor solution which is a mixture of isopropanol and ZrCl_4_ through spin coating; (**c**) simultaneous removal of PS NPs and calcination of ZrO_2_ through heat treatment; (**d**) thermal evaporation of Au nanoparticles onto the already-made *p*ZrO_2_ substrate.

Uniform PS NPs, with a diameter of 165 nm that were synthesized through surfactant-free emulsion polymerization, were first deposited onto a pre-cleaned 2 × 2 cm^2^ Si substrate Si by drawing Si out of PS NPs suspended in water, as shown in Figure 1a. The PS-coated Si was then subjected to slight annealing at 50 °C for 10 min, ensuring that all of the solvents has been evaporated before spin-coating the precursor ZrO_2_ solution onto Si as illustrated in Figure 1b. The precursor solution was prepared by dissolving pre-weighed zirconium tetrachloride (ZrCl_4_, 98%, Acros Organics, Pittsburgh, PA, USA) in isopropanol (99.8%, Panreac AppliChem, Darmstadt, Germany) that was left to stand for 24 h in a sealed vessel until it achieved a gel-like consistency. Subsequently it was then subjected to heat treatment at 600 °C for 3 h at a heating rate of 5 °C/min, for complete removal of the template layer of PS NPs and calcination of the remaining ZrO_2_, leaving behind an ordered concave structure of ZrO_2_ on Si, denoted as *p*ZrO_2_, as shown in Figure 1c. The size and dimension of PS NPs, the concentration of the precursor solution, the parameters of spin coating procedure, and the temperature for calcination were all held constant throughout the experiment. As briefly illustrated in Figure 1d, Au NPs were then deposited onto *p*ZrO_2_ through thermal evaporation using an electron beam evaporator (VT1-10CE, ULVAC, Chigasaki, Japan). The deposition was performed under a pressure of 6~8 × 10^−6^ torr, having a deposition rate of 0.1 Å/sec at varying deposition thickness of 1.5, 2.0, 3.0, and 5.0 nm. The *p*ZrO_2_ containing Au NPs in a porous structure was prepared, and denoted as Au NPs (*x*)/*p*ZrO_2_, where x indicates the deposition thickness of Au NPs in nm.

### 2.2. Physical Characterization of Au NPs (x)/pZrO_2_

The morphology and composition of Au NPs (x)/*p*ZrO_2_ substrates were analyzed through high-resolution thermal field emission scanning electron microscope, coupled with energy dispersive X-ray spectroscopy (FE-SEM/EDS, JSM-7000, JEOL, Tokyo, Japan) and atomic force microscope (AFM, Dimension Icon, Bruker, Karlsruhe, Germany) in dynamic mode (scan rate of 0.5 Hz, Au front coating (W_Au_ = 5.18 eV)). Photo-images of Au NPs (*x*)/*p*ZrO_2_ were taken by FE-SEM using secondary electron imaging mode at an accelerating voltage of 10 kV. The size and dimension of Au NPs were then measured on the resulting images with the use of ImageJ software (version 1.47, National Institutes of Health, Rockville, MD, USA).

### 2.3. SERS Property of Au NPs (x)/pZrO_2_

The as-fabricated Au NPs (*x*)/*p*ZrO_2_ was then subjected for evaluating their SERS properties through Raman spectrometer (Renishaw, Wotton-under-Edge, UK) with an incident power of 3 mW and an air-cooled charge-coupled device (CCD) as the detector. All measurements were done in a dry environment at ambient temperature, wherein a fixed amount of the test probe solution of Rhodamine 6G (R6G) at varying concentrations (10^−2^ M down to 10^−7^ M) was dropped onto the reference Si and Au NPs (*x*)/*p*ZrO_2_, respectively. The substrates were left to dry before subjecting to experiments. The Raman spectrometer was set up in a way that the objective of the optical microscope was set to 50X, the spot size of Raman laser fixed at a diameter of 1 µm, and the diode and He-Ne lasers at excitation wavelengths of 633 and 785 nm with gratings at 1800 lines/nm and 1200 lines/nm, respectively. The calibration of the spectrum was done first before taking any measurements with the use of a standard Si and at a laser power set at 100%. The obtained Raman spectra were processed in to remove background fluorescent signals and noise that may have been generated from the use of an objective lens to focus the laser spot. Baseline correction and smoothing were also deemed necessary to normalize the data for acquiring more defined signals.

### 2.4. Trace Analysis of Pesticide(s) upon Au NPs (x)/pZrO_2_

The optimized Au NPs (*x*)/*p*ZrO_2_ was employed for their efficacy in detecting trace amounts of pesticide(s), which both phosmet and carbaryl (Sigma Aldrich; pesticides in powder form, St. Louis, MO, USA) were used. Standard solutions from 10^−2^ to 10^−6^ M were prepared by dissolving the pesticides in analytical grade ethanol, while equimolar mixtures of both pesticides at 10^−2^, 10^−3^, and 10^−4^ M were also prepared. The effect of SERS involving pesticide solutions was performed in the same manner as those with R6G solutions wherein the solutions were dropped onto the optimized Au NPs (*x*)/*p*ZrO_2_ and were allowed to dry at room temperature.

## 3. Results and Discussion

### 3.1. Size and Dimension of Au NPs (x)/pZrO_2_

**Figure 2:**
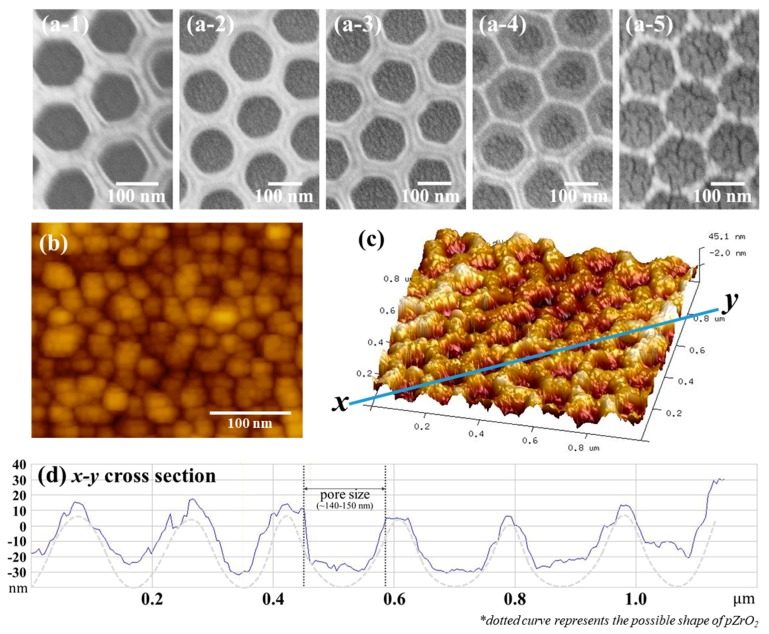
Surface morphologies of the fabricated Au NPs/*p*ZrO_2_: (**a**) SEM images from left to right—*p*ZrO_2_, Au NPs (1.5)/*p*ZrO_2_, Au NPs (2.0)/*p*ZrO_2_, Au NPs (3.0)/*p*ZrO_2_, and Au NPs (5.0)/pZrO_2_ shows the morphological evolution of the substrate in terms of Au NP deposition thickness; (**b**) 2D AFM image shows the estimated sizes of the Au NPs; (**c**) 3D AFM image of Au NPs (3.0)/*p*ZrO_2_ clearly shows the presence of porous cavities as formed by the ZrO_2_; (**d**) Cross-sectional profile of the structure corresponding to the x-y line in Figure 2c confirms the structure of the porous ZrO_2_.

Scanning electron microscope (SEM) images and size distribution of the PS NPs and ZrO_2_ pores are provided in Figure S1. In Figure 2a, the morphologies of Au NPs (*x*)/*p*ZrO_2_, *x* = 0 (a-1), 1.5 (a-2), 2.0 (a-3), 3.0 (a-4), and 5.0 (a-5) were demonstrated. The photo-images showed that when x = 2.0 (relative loose) and 3.0 (relative dense), Au NPs were relatively appropriate to cover upon *p*ZrO_2_. With increasing deposition thickness (*x*), Au NPs appeared to increase in quantity and eventually exhibited agglomeration, as shown in *x* = 5.0 nm. In Figure 2b, inside a one-unit structure of Au NPs (3.0)/*p*ZrO_2_, Au NPs exhibited elongated or elliptical in shapes as normally produced through thermal evaporation at low deposition thickness conditions [23].

In Figure 2c, an example of AFM topographical image (1 µm × 1 µm) from Au NPs (3.0)/*p*ZrO_2_ showed the presence of uniform concave-like cavities on the substrate. As illustrated by a cross-sectional profile (x-y cross section), in Figure 2d, the concave cavities exhibited a geometry with depths ranging around 50 nm, whereas the width of the concave structure was estimated to be 140~150 nm. As compared the diameter (around 165 nm) of PS NPs, the shrinkage of width and depth was most probably due to the addition of ZrO_2_, followed by the removal of PS NPs and the deposition of Au NPs on the *p*ZrO_2_.

In Figure 2b, inside a cavity of Au NPs (3.0)/*p*ZrO_2_, the sizes and dimensions (i.e., lateral and longitudinal) of Au NPs exhibited diameters ranging from 15~50 nm (i.e., with the depths of upper or lower layers), and averaged around 25 nm, while the individual NPs in elongated or elliptical shapes showed closely collective (or sufficiently covered) within *p*ZrO_2_. Note that as the target species is included in Au NPs (*x*)/*p*ZrO_2_, followed by the incident Raman laser, an effect of collective amplification that increases surface contacts near Au-Au “hot spots” is thus anticipated. Among them, Au NPs (3.0)/*p*ZrO_2_ with a relative dense Au NPs in the concave pore of *p*ZrO_2_ is preferably selected. Au NPs appearing as clumping the individual NPs owing to an increased amount (e.g., *x* = 0.5) of material [24] is relatively inappropriate for the design.

### 3.2. SERS Property of Au NPs (3.0)/pZrO_2_

**Figure 3:**
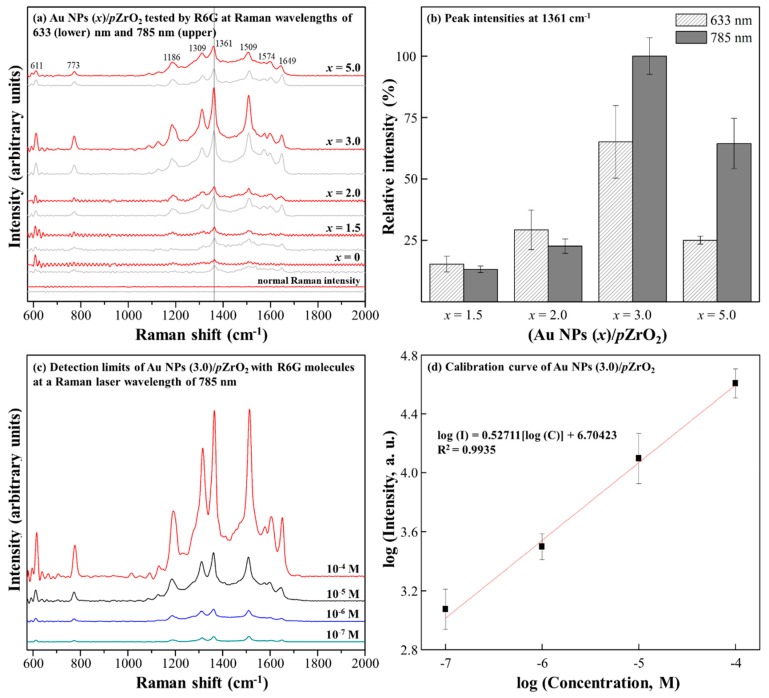
(**a**) SERS spectra of 10^−3^ M R6G measured at the respective excitation wavelengths of 633 and 785 nm on Au NPs/*p*ZrO_2_ SERS substrates and normal Raman on plain Si; indicated as shown is the most intense peak which is at 1361 cm^−1^; (**b**) Relative Raman intensities of 10^−3^ M R6G at the 1361 cm^−1^ peak taken using the Au NPs (*x*)/*p*ZrO_2_ samples at 633 and 785 nm excitation wavelengths show the effects of varying the laser wavelengths and Au NP deposition thickness; (**c**) SERS spectra of varying concentrations of R6G on Au 3.0/*p*ZrO_2_ showing a relative decrease in the peak intensities with decreasing concentration; (**d**) Calibration plot produced by the logarithmic linear trend between the 1361 cm^−1^ peak intensity and concentration.

The relationship between the overall SERS effect with Au NPs (*x*)/*p*ZrO_2_ was further investigated by checking their Raman intensities with the test molecule probe, R6G, with the variations of x and the excitation wavelengths (i.e., 633 and 785 nm) of Raman laser. As shown in Figure 3a, for both laser wavelengths, there is a noticeable change in the Raman intensities with varying deposition thickness (*x*) of Au NPs. In Figure 3b, considering the most intense characteristic peak of R6G at 1361 cm^−1^ as the reference, for both laser wavelengths, the highest intensities were acquired with the use of Au NPs (*x*)/*p*ZrO_2_ at *x* = 3.0 or 5.0 (i.e., relatively abundant Au NPs in both cases). However, Au NPs (3.0)/*p*ZrO_2_ provided a better combination among Au-Au “hot spots”, R6G molecules, and Raman laser wavelength of 785 nm within the concave pore of *p*ZrO_2_, an optimal condition was therefore chosen. Reproducibility of signals was investigated by obtaining the R6G SERS spectra from 6 randomly chosen spots across one sample of Au NPs (3.0)/*p*ZrO_2_, as shown in Figure S2.

In Figure 3c, based on the above result, a detection limit for R6G molecule with the concentrations of 10^−4^, 10^−5^, 10^−6^, and 10^−7^ M was examined. In Figure 3d, by taking 1361 cm^−1^ as the indication, a linear log relation between Raman intensity and the concentration was recorded, and a very low detection limit of 10^−7^ M was estimated. In some studies, a quantitative determination for a trace amount of a target analyte in a sample has been done [25,26]. The EF of Au NPs (3.0)/*p*ZrO_2_ is calculated to be 7.0 × 10^7^, typically comparable to an averaged EF of recently-developed Au/oxide composite as SERS substrate [27,28], in particular with Au/ZrO_2_ nanofiber substrate that exhibited an EF of 10^7^ [28].

For SERS effects imparted by the individual component of Au NPs or *p*ZrO_2_, in comparison to that of combined Au NPs/*p*ZrO_2_ composite, their SERS spectra were shown in Figure S3. The *p*ZrO_2_ alone exhibited weak SERS intensity as observed through the enhancement of the major characteristic peaks of R6G, with an average EF of ~10^2^, attributing to the chemical mechanism that works between R6G and ZrO_2_. Among the above substrates in Figure S1, Au NPs (3.0)/*p*ZrO_2_, exhibited much significant enhancement that takes the benefits from the individual components and the combination of both. The inset shows that the particular peak at 1311 cm^−1^ with Au NPs (3.0)/*p*ZrO_2_ is shifted when Au NPs (3.0)/Si is used, whereas it becomes absent (or not enhanced) in the spectrum produced in *p*ZrO_2_. It is likely that peaks shifting or absence are resulting from the variation of metal-oxide interactions or that of molecular orientations with respect to the substrate composition [29].

### 3.3. SERS Properties of Au NPs (x)/pZrO_2_ to Pesticides Detection

**Figure 4:**
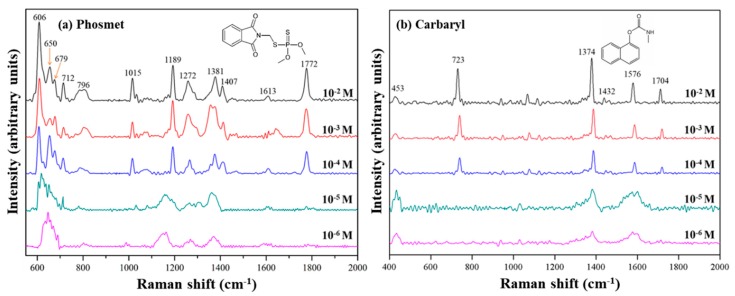
SERS spectra of standard solutions of (**a**) phosmet and (**b**) carbaryl at varying concentrations on Au NPs (3.0)/*p*ZrO_2_.

To evaluate the effect of SERS upon Au NPs (3.0)/*p*ZrO_2_, two pesticides of different chemical structures were respectively examined. In Figure 4a,b, the characteristic peaks of Phosmet and Carbaryl with different concentrations were shown. As summarized in Table S1, the peaks at 606, 653, 675, 712, 796, 1014, 1189, 1260, 1381, 1409, and 1772 cm^−1^ are represented for Phosmet, while those at 713, 1380, 1441, and 1582 cm^−1^ for Carbaryl [30,31,32,33]. For both pesticides, at 10^−2^, 10^−3^, and 10^−4^ M, the characteristic peaks appeared to be identical. At lower concentrations of 10^−5^ and 10^−6^ M, the effect of SERS became less distinctive.

By taking the characteristic peaks at 606 and 1189 cm^−1^ for Phosmet and those at 1062 and 1576 cm^−1^ for Carbaryl, the SERS signal of the pesticides was still detectable, therefore, Au NPs (3.0)/*p*ZrO_2_ was competent to have the detection limit lower than 10^−6^ M. According to the regulations set by the Food and Agriculture Organization of the United Nations (FAO) and World Health Organization (WHO), the maximum residue limit for both pesticides in crop products is defined around 5~10 ppm, or 10^−5^ to 10^−4^ M, as a consequence, Au NPs (3.0)/*p*ZrO_2_ is presumably used for trace detection of the measured pesticides.

However, it should be noted that in this study, only standard solutions were used and the effects in simulated and real conditions would differ from the presented results. It was also observed that for both pesticides at lower concentrations (10^−5^ to 10^−4^ M), major peaks appear to broaden possibly due to the background of the substrate affecting the resulting Raman spectra. Nevertheless, for both Phosmet and Carbaryl, major characteristic Raman peaks appear regardless of concentration.

### 3.4. Particular Two Pesticides Detection Using Au NPs (3.0)/pZrO_2_

**Figure 5:**
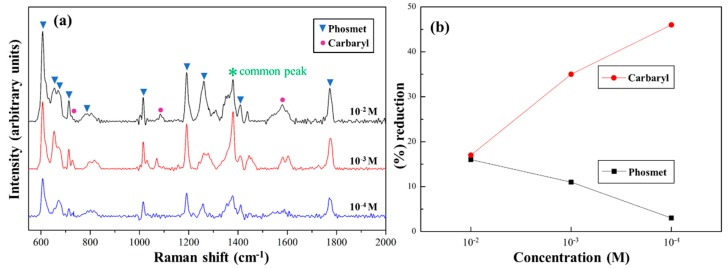
(**a**) Average SERS spectra of equimolar mixtures of phosmet and carbaryl at varying concentrations show that Au NPs (3.0)/*p*ZrO_2_ is capable of multiplex detection; (**b**) Plot of the % reduction (from standard solution to mixture condition) in the peak intensity ratios obtained from the two major characteristic peaks of each pesticide.

Furthermore, SERS-active substrate of Au NPs (3.0)/*p*ZrO_2_ was used to perform the detection of both pesticides in a single run. In Figure 5a, the peaks found in the resulting SERS spectra are relevant to both pesticides, indicating that the substrate is capable of detecting analytes. As with any solution of mixtures, the involved analytes tend to compete for adsorption sites on the substrate, thus further investigation that influences the measured signals or intensities is necessary. In Figure 5a, two strongest peaks from each pesticide (606 and 1189 cm^−1^ for Phosmet and 1062 and 1576 cm^−1^ for Carbaryl) were taken, and the intensities of the peaks for each pesticide were used to obtain ratios (i.e., I_606_/I_1189_ for Phosmet and I_1062_/I_1576_ for Carbaryl) from both standard solution and mixture conditions. The peak intensity ratios in the mixture condition were apparently lower than that in each standard solution condition. 

In Figure 5b, this decrease is represented by the percentage (%) reduction. These two pesticides showed diversified into opposite trends in terms of the % reduction of peak intensity ratios with concentrations: At a higher concentration of 10^−2^ M, % reductions for both pesticides were comparable, whereas the differences enlarged as the concentrations decreased. In Figure 5a, with the decrease of mixture concentrations, the peaks corresponding to Phosmet are relatively stronger than those to Carbaryl, as also shown low % reduction in Figure 5b. It indicates that despite being in a multiple analyte environment, the relative peak intensities of Phosmet are sufficiently strong to be detected. Phosmet has a strong binding affinity to the substrate owing to its sulfur and organophosphate components, which bind to Au and ZrO_2_ components (i.e., the hot spots) of the substrate, respectively. This strong affinity allows a sufficient number of Phosmet molecules even at low concentrations to be adsorbed and detected.

In contrast to Phosmet, Carbaryl suspends within Au NPs (3.0)/*p*ZrO_2_, relatively away from Au-Au hot spots or ZrO_2_ surfaces. Therefore, considering the difference in the affinities of the pesticides to the substrate, Carbaryl was shown to exhibit a greater % reduction. This is notably due to the quenching of the Carbaryl signals brought by competitive adsorption among the analytes wherein the presence of Phosmet in the mixture takes up the majority of the available adsorption sites, due to its strong affinity to the substrate [34]. The high selectivity of Au NPs (3.0)/*p*ZrO_2_ towards Phosmet as demonstrated in this study aligned with the results of another study that also utilized a substrate of Au/ZrO_2_ with a different morphology to detect 4 various pesticides in a mixture [28]. In Figure 4, each of both pesticide solutions presented a promising SERS result; however, it was not proportional in the case of the mixture condition owing to the changes of the chemical environment. Consequently, for Au NPs (3.0)/*p*ZrO_2_, the affinity of target molecules to the “hot spots” area should be considered when performing multiple detections of analytes through the technique of SERS.

Chemical enhancement mechanism is based on the modification of the electronic properties of the adsorbed analyte, in which the vibrations resulting from the electronic transitions improve the effect of SERS by 1-2 orders of magnitude [8]. In this study, Phosmet having a stronger affinity to the substrate would thus have a more pronounced chemical enhancement effect, as compared to the weakly-bonded Carbaryl. With this fact, Au NPs (3.0)/*p*ZrO_2_ has not only shown the capability to detect trace amounts of various pesticides but has also exhibited selectivity specific to organophosphate pesticides.

### 3.5. Particular Two Pesticides Detection Using Au NPs (3.0)/pZrO_2_

**Figure 6:**
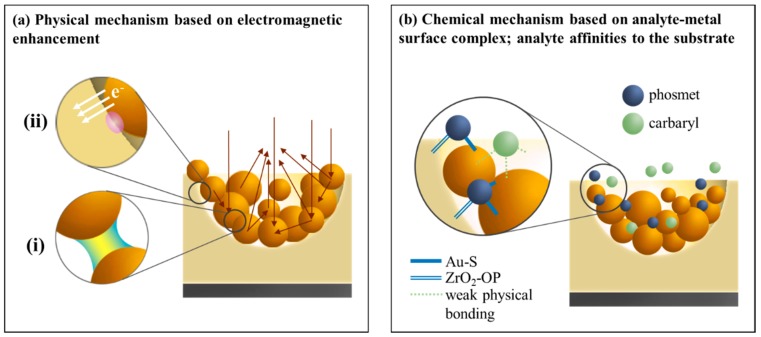
Proposed mechanisms that provide the overall SERS amplification: (**a**) Physical mechanism of the Au/*p*ZrO_2_ substrate showing two major contributors: (i) Electromagnetic enhancement brought by the formation of hot spots between Au NPs. Additionally, the arrows indicate the convergent scattering of light with respect to the arrangement of Au NPs in a concave cavity, and (ii) interfacial charge transfer between Au NPs and ZrO_2_, and (**b**) Chemical mechanism owing to the formation of analyte-substrate complexes, related to the affinities of the analyte molecules to the substrate.

Physical and chemical enhancement mechanisms involved in the effect of SERS imparted by Au NPs/*p*ZrO_2_ are shown in Figure 6. In Figure 6a, the physical mechanism is largely dependent on the substrate material and geometry. In terms of substrate geometry, two aspects can be considered: Firstly, since Au NPs follow an arrangement according to the concave shape of the pore, under a vertical incident laser, Au NPs may direct the light to converge at the center of the pores, as indicated by the arrows in Figure 6a. More hot spots for SERS are generated from the overlaps of electric field and brought by the negative curvature arrangement of Au NPs. Second, the concave pore of ZrO_2_ provides an increase of surface area for Au NPs to be adsorbed on, compared to that when Au NPs are on a flat surface with the same projected horizontal area.

Looking into a smaller scale, Figure 6a(i) shows the dominant electromagnetic mechanism provided by the hot spots formed in the gaps between Au NPs as a result of LSPR coupling. In addition, Figure 6a(ii) shows the transfer of charges between Au NP and ZrO_2_, a low Schottky barrier between these components eases the charge transfer, resulting in an effect of SERS. This transfer results to concentrating the plasmon-induced electromagnetic field at the metal/semiconductor interface, which provides enhancement not only in generating effective carriers in the plasmonic component but also induces further charge transfer, resulting in an overall enhancement in the electric field and SERS signals.

On the other hand, the chemical mechanism is largely analyte-dependent. In Figure 6b, both Phosmet and Carbaryl can be adsorbed onto Au NPs (*x*)/*p*ZrO_2_ in different degrees, while an analyte-substrate complex is formed that modifies the polarizability of as-adsorbed molecules. However, since Phosmet possesses a higher affinity to the substrate, the tendency of forming a strong complex with the substrate would be higher than that of Carbaryl, which could only form weaker complexes and lead to be even unable to modify the electronic structure of Carbaryl. Therefore, this as well explains the fact that Au NPs (*x*)/*p*ZrO_2_ is selective towards Phosmet.

Both mechanisms described in Figure 6a,b simultaneously function when the substrate system is subjected to Raman spectroscopy, as a consequence, the combined effects from both mechanisms bring a synergistic effect with collective SERS amplification that provides a strengthened capability of Au NPs (*x*)/*p*ZrO_2_ for the application of trace detection.

## 4. Conclusions

A substrate specially designed for collective SERS amplification by taking into consideration substrate geometry and materials, as well as its interactions with target analytes, was presented in this study. Au NPs were deposited onto a concave-pore ZrO_2_ array, and the optimized substrate Au NPs (3.0)/*p*ZrO_2_ provided an EF of 7.0 × 10^7^, which was competent to detect trace amounts of Phosmet and Carbaryl at a detection limit of 10^−6^ M. Moreover, multiplex detection of both pesticides was demonstrated as well but the substrate exhibited a higher selectivity towards Phosmet, due to its functional groups having a strong affinity towards both Au and ZrO_2_ components in/on the substrate. The combined effects of the physical mechanisms involving the geometry of the substrate of Au NPs arranged in concave ZrO_2_ pores, Au-Au hot spots, and Au-ZrO_2_ charge transfers, and chemical mechanisms associated with the interactions between the pesticide molecules and the substrate, were thus proposed to be the contributing factors behind the collective SERS amplification provided by the substrate.

## Supplementary Materials

The following are available online at https://www.mdpi.com/2079-4991/9/5/664/s1, Figure S1: SEM images of (a) polystyrene nanoparticles and (b) porous ZrO_2_ arranged in a monolayer, and the (c,d) corresponding size distribution histograms, Figure S2: SERS intensity plots taken from 6 positions at 3 different peaks (left) and the corresponding full-range spectra (right) of 10^−3^ M R6G, Figure S3: SERS spectra of R6G on different substrates (bare *p*ZrO_2_, Au 3.0 on Si, and Au NPs (3.0)/*p*ZrO_2_) show that each individual component exhibit SERS activity, inset shows the highlighted region in the plot wherein there is an observed shift in peaks, Table S1: Major characteristic peaks of phosmet and carbaryl and their corresponding vibrational modes [19,28,30,31,32,33].
