# Supplementary material for: SERS-Active Substrate with Collective Amplification Design for Trace Analysis of Pesticides
Source: Nanomaterials (Basel). 2019 Apr 27;9(5):664. doi: 10.3390/nano9050664 (PMC6566408; doi:10.3390/nano9050664)
Supplement: Supplementary file 1 [file nanomaterials-09-00664-s001.pdf]

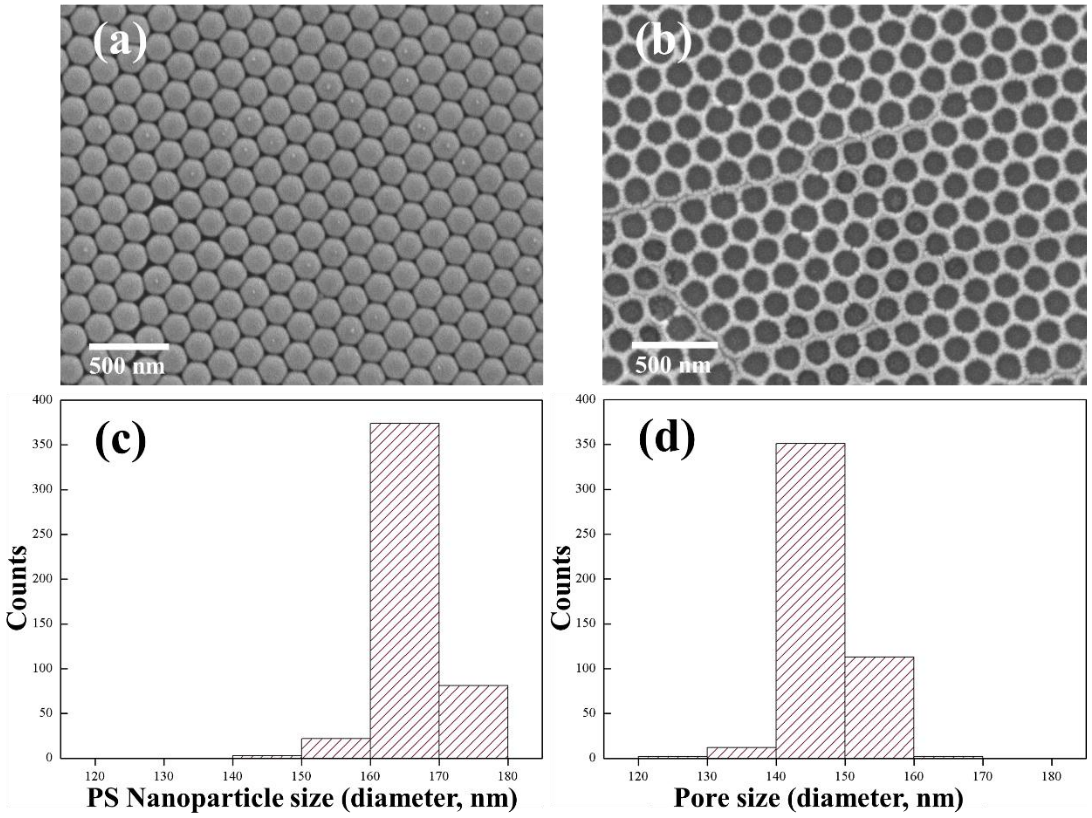

Figure 1. SEM images of (a) polystyrene nanoparticles and (b) porous ZrO_2 arranged in a monolayer, and the (c,d) corresponding size distribution histograms.

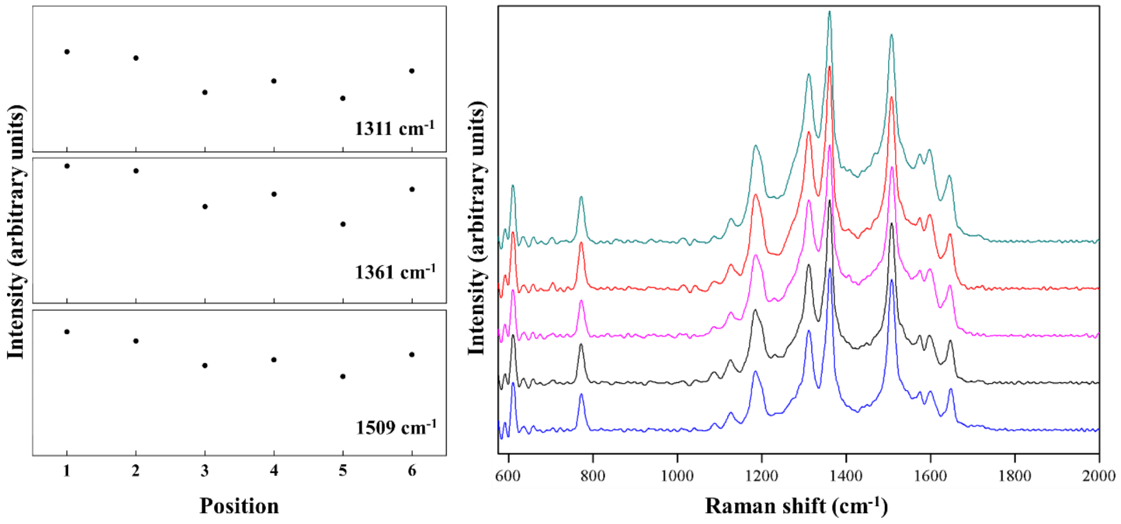

Figure S2: SERS intensity plots taken from 6 positions at 3 different peaks (left) and the corresponding full-range spectra (right) of 10^{-3} M R6G.

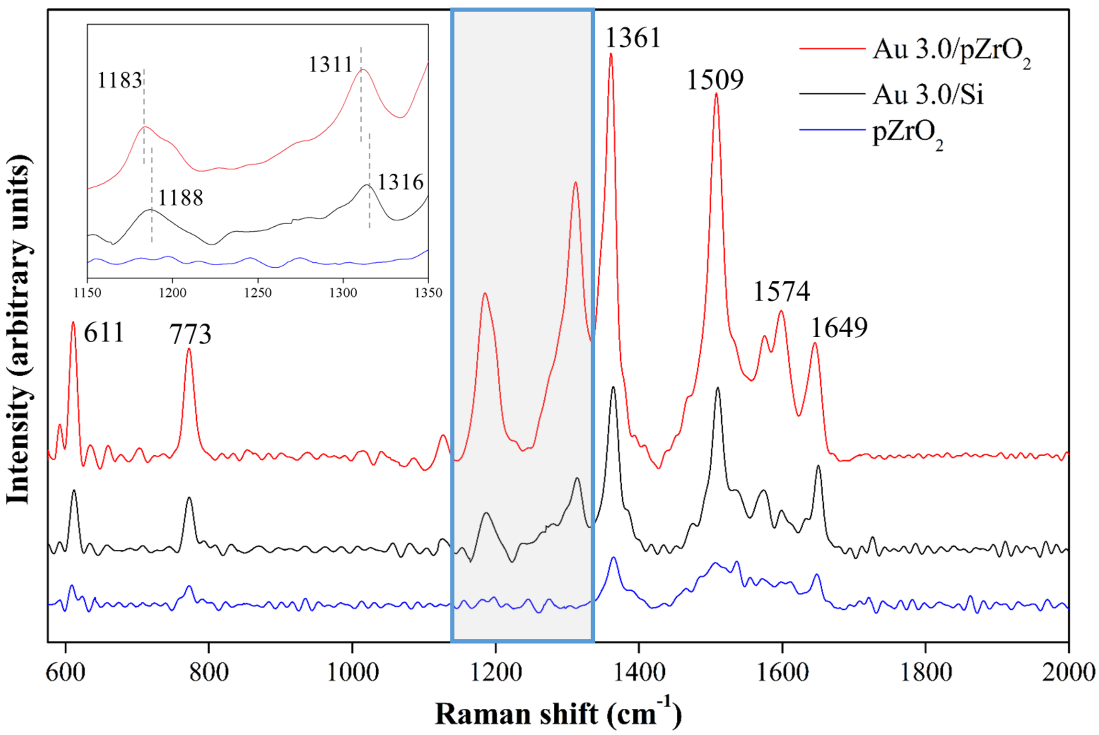

Figure S3: SERS spectra of R6G on different substrates (bare *p*ZrO_2, Au 3.0 on Si, and Au NPs (3.0)/*p*ZrO_2) show that each individual component exhibit SERS activity, inset shows the highlighted region in the plot wherein there is an observed shift in peaks.

Table S1. Major characteristic peaks of phosmet and carbaryl and their corresponding vibrational modes [19,28,30–33].

Pesticide	Raman shift (cm^{-1})	Assignment
Phosmet	606	δ(C=O), in-plane deformation vibration
	653	δ(P=S), in-plane deformation vibration
	675	υ(P=S), stretching
	712	benzene ring breathing
	796	υ(P–O) + δ(CH_3)
	1014	asymmetric stretching of P–O–C deformation
	1189	δ(C–N), in-plane deformation vibration
	1260	υ(C–N), stretching in S–CH_2–N
	1381	δ(CH_3), in-plane deformation vibration
	1409	γ(C–H), out-of-plane deformation vibration
	1772	υ(C=O), stretching
Carbaryl	713	δ(NCOC), in-plane deformation vibration
	1380	symmetric ring vibration
	1441	ω(C–H), non-planar rocking
	1582	υ(C=C), stretching in naphthalene ring
